# Challenges in Developing a Microglial NF-Kappa B Conditional Mouse Model for Glioblastoma Studies

**DOI:** 10.3390/cells15181668

**Published:** 2026-09-16

**Authors:** Amy T. Trang, Ahmet Alptekin, Michael Goodall, Surendra K. Rajpurohit, Ryan Frerichs, Ashley Koch, Thaiz F. Borin, Ahmet K. Korkaya, Karan Soni, Ali S. Arbab, Jennifer W. Bradford

**Affiliations:** 1 Department of Biological Sciences, Augusta University, Augusta, GA 30912, USA; 2 Georgia Cancer Center, Medical College of Georgia, Augusta University, Augusta, GA 30912, USA; 3 Department of Biochemistry and Molecular Biology, Medical College of Georgia, Augusta University, Augusta, GA 30912, USA

**Keywords:** glioblastoma, NF-κB, microglia

## Abstract

Tumor microenvironments (TME) play a crucial role in the formation, progression, and drug resistance of many cancers, including glioblastoma (GBM). Microglia are the resident innate immune cells of the central nervous system and are associated with brain cancers like GBM. However, there are limited studies about the specific effects that microglia have on GBM tumors. One possible way to reduce GBM tumor progression is by targeting key signaling pathways in microglia, such as the nuclear factor-kappa B (NF-κB) signaling pathway, which has been implicated in both microglia/macrophage phenotype polarization and in brain cancers like GBM. Therefore, this study aimed to produce a p65fl/fl/CX3CR1creER/+ mouse model, an inducible p65 knockout mouse model to be used for studying how inhibition of canonical NF-κB signaling in microglia affects GBM tumors. Following tamoxifen administration to the mouse, p65, a transcription factor of the canonical NF-κB pathway, should be deleted in microglia due to activation of a Cre recombinase (creER) under the control of the CX3CR1 promoter. This mouse model was characterized to determine whether the *p65* gene was effectively deleted in microglia using this system. Our data show that tamoxifen-treated p65fl/fl/CX3CR1creER/+ mice had only partial deletion of the *p65* gene, which corresponded to only slightly lower p65 expression in microglia compared to control mice. Partial p65 deletion in microglia was also observed in p65fl/fl/CX3CR1creER/+ mice that received both GBM implantations and tamoxifen treatment compared to a vehicle control group. Despite the low p65 deletion efficiency, we show that slight decreases in microglial p65 can alter gene expression of various genes involved in microglial phenotype polarization, which is exacerbated in the context of GBM. Overall, the data from this study show minimal *p65* deletion in microglia from tamoxifen-treated p65fl/fl/CX3CR1creER/+ mice. The limitations of this model will serve as guidance to the GBM TME field for future animal model production.

## 1. Introduction

Glioblastoma (GBM), classified as a grade IV astrocytoma by the World Health Organization, has an extremely poor prognosis despite the use of various treatment methods including surgical resection, temozolomide chemotherapy, radiation therapy, and combinations of these treatments [1,2,3]. In addition, these standard therapies often cause undesirable side effects, tissue damage, or toxicity in patients, which prompts the need to develop safer, yet effective, treatment methods [3].

GBM tumors have dynamic, heterogeneous tumor microenvironments (TME) [1], which impact the formation, progression, and drug resistance of GBM [4]. GBM TMEs are composed of a variety of cell types, which complicate the treatment strategy, as each cell type can respond differently to a specific treatment [1]. Tumor-associated macrophages (TAMs) and microglia infiltrate the GBM tumor and can make up 40% of the TME [5,6]. TAMs are peripheral macrophages, which are derived from bone marrow myeloid cells [4], whereas microglia are myeloid cells derived from the embryonic yolk sac [7]. Both immune cells can polarize into a classical, tumoricidal (M1) or an alternative, tumor-promoting (M2) state depending on their environment and other factors [4]. Through the secretion of various cytokines, TAMs can enhance neovascularization, tumor growth, promote an epithelial-mesenchymal transition (EMT) in cancer cells, and induce cancer cell metastasis [4,8], exemplifying how cells in the TME can favor tumor growth and progression.

Given the importance of interactions within the TME, targeting key signaling pathways involved in sustaining GBM tumors could be an effective approach to reduce tumor progression and prolong the life of the patient. One specific signaling pathway implicated in different types of cancer, including GBM, is the nuclear factor-kappa B (NF-κB) signaling pathway [1]. NF-κB helps regulate critical cellular processes such as apoptosis, inflammation, cancer progression, and cell proliferation [1,9]. Abnormally high NF-κB activity has been identified in various cancers in addition to autoimmune diseases, which makes this pathway an important target [1,9,10]. The NF-κB family is composed of five transcription factors: RelA/p65 (referred to as p65 from here on), RelB, c-Rel, p50 (p105 precursor), and p52 (p100 precursor) [9], which are part of either the non-canonical or canonical NF-κB pathways [9]. Importantly, canonical NF-κB activity is associated with maintaining the tumor-promoting M2 phenotype of TAMs [11].

Some studies have reported that inhibition or deletion of essential proteins in the canonical NF-κB pathway reduces tumor size and growth for different cancers. According to Hagemann et al., inhibition of IKKβ, and therefore canonical NF-κB signaling, in TAMs and bone marrow-derived macrophages (BMDMs) exerted an anti-tumor effect on ovarian tumors [11]. Moreover, Bredel et al. suggest that inhibition of the canonical NF-κB pathway through re-expression of the *NFKBIA* gene, which encodes the IκBα protein that sequesters the p50:p65 heterodimer, reduces tumor progression and prolongs patient survival [10]. Furthermore, our research in collaboration with Dr. Arbab’s group at the Georgia Cancer Center has shown that deletion of p65 in murine myeloid cells reduced GBM tumor growth, decreased tumor cell proliferation, and increased apoptosis of tumor cells [2]. It is unclear whether inhibiting NF-κB signaling in microglia might show these same effects.

To study the role of canonical NF-κB signaling in microglia, we have generated a novel p65^fl/fl^/CX_3_CR1^CreER/+^ transgenic mouse model, which targets the p65 transcription factor in microglia. The CX_3_ chemokine receptor 1 (CX_3_CR1) promoter allows Cre recombinase to be expressed in mononuclear phagocytes like microglia, monocytes, macrophages, dendritic cells, and natural killer cells, preventing the deletion of the *p65* gene in other cell types [12,13,14]. Due to the slow turnover rate of microglia compared to other mononuclear phagocytes, microglia should maintain the *p65* gene deletion, whereas the other mononuclear phagocytes will eventually reestablish p65 expression [13,14]. This model is inducible and conditional as it uses a Cre recombinase under the control of the estrogen receptor (CreER). Once tamoxifen-activated Cre recombinase binds to the loxP sites in the *p65* gene, it performs a recombination event involving the excision of the floxed gene from the genome [12,15]. This results in a nonfunctional gene product and, therefore, should render the NF-κB pathway nonfunctional. In this study, we generated and characterized the p65^fl/fl^/CX_3_CR1^CreER/+^ (p65KO) transgenic mouse model to determine whether the floxed *p65* gene is deleted from the genome in mouse microglia. Despite various tamoxifen treatment regimens, our model only achieved partial deletion of the *p65* gene in microglia. This study provides additional evidence of the challenges seen by others in producing an effective microglial NF-kappa B knockout model.

## 2. Materials and Methods

### 2.1. Mouse Genotypes

The following mouse genotypes used were p65^fl/fl^ mice [16], B6.129P2(C)-*Cx3cr1^tm2.1(cre/ERT2)Jung^*/J mice (CX_3_CR1^CreER^; The Jackson Laboratory, strain #020940, Bar Harbor, ME, USA), p65^fl/fl^/CX_3_CR1^CreER/+^ (p65KO) mice, B6.129P2-*Lyz2^tm1(cre)Ifo^*/J (LysMCre; Jackson Laboratory; strain #004781), and p65^fl/fl^LysMCre mice [17]. All strains have a C57BL/6 background. To generate the p65^fl/fl^/CX_3_CR1^CreER/+^ mice, p65^fl/fl^ mice were crossed to CX_3_CR1^CreER^ mice until homozygous progeny were obtained. p65^fl/fl^, CX_3_CR1^CreER^ and LysMCre mice were used as controls. The care and use of these animals was approved by the Institutional Animal Care and Use Committee (IACUC) at Augusta University. Experiments were performed in strict accordance with the guidelines set by the IACUC at Augusta University and by the NIH.

### 2.2. Genotyping PCR-p65 LoxP PCR

Genotypes were confirmed via PCR analysis using our established tissue lysis and ethanol DNA extraction method [17]. For genotyping CX_3_CR1 CreER mice, the following primers were used: wild-type primer (IDT; 5’-AGCTCACGACTGCCTTCTTC-3’, 0.16 µM), common primer (IDT; 5’-ACGCCCAGACTAATGGTGAC-3’, 0.5 µM), mutant primer (IDT; 5’-GTTAATGACCTGCAGCCAAG-3’, 0.6 µM). PCR conditions were 1 denaturation cycle at 94 °C for 2 min, 11 cycles of denaturation at 94 °C for 20 s, annealing at 65 °C for 15 s (with a 0.5 °C decrease per cycle), and elongation at 68 °C for 10 s. This was followed by 29 cycles of denaturation at 94 °C for 15 s, annealing at 60 °C for 15 s, elongation at 72 °C for 10 s, and an elongation cycle at 72 °C for 2 min. DNA band(s) of the wild-type *Cre* gene (151 bp), mutant *Cre* gene (230 bp), or both genes (heterozygous) were observed via gel electrophoresis.

### 2.3. GL261 Cell Culture

Syngeneic mouse glioma 261 GBM (GL261) cells were gifted by Dr. Theodore Johnson of the Georgia Cancer Center. These cells were confirmed to be mycoplasma-free (ATCC; 30-1012K). The GL261 media consisted of RPMI Medium 1640 (Gibco, Thermo Fisher Scientific, Grand Island, NY, USA; MT10040CV), 10% FBS, 1% penicillin–streptomycin, and L-glutamine. Cells were maintained at 37 °C with 5.0% CO_2_.

### 2.4. Tamoxifen Treatment Schedule and GBM Tumor Implantation

Five- or six-week-old mice were given tamoxifen dissolved in corn oil or corn oil control via oral gavage to activate the CreER or as a control, respectively. Tamoxifen-free base (Sigma-Aldrich, St. Louis, MO, USA; T5648) was dissolved in warm corn oil (Sigma-Aldrich, St. Louis, MO, USA; C8267).

Two different treatment schedules were tested to optimize *p65* gene deletion prior to GBM implantation. Two doses of 5mg tamoxifen or corn oil were given to eight p65^fl/fl^CX_3_CR1^CreER/+^ mice via oral gavage with a three-day interval between each dose. Four days following the second dose, 10,000 syngeneic GL261-Luc cells in 2 µL of serum-free RPMI were implanted intracranially into the right cerebral hemisphere of each mouse using the Stoelting mouse stereotactic device [18]; 1× PBS was injected as the control. One week following GBM implantation or PBS injection, mice were given two additional doses of 5mg tamoxifen or corn oil via oral gavage, with a two-day interval between each dose. Bioluminescent images were taken of the treated mice to observe GBM tumor formation using the Ami X imaging system (Spectral Instruments Imaging, Tucson, AZ, USA) (via 3 mg intraperitoneal injection of luciferin). Tumors or brain tissue were harvested one week later for flow cytometry.

For the second treatment schedule, two doses of 5mg tamoxifen or corn oil control were administered to three p65^fl/fl^CX_3_CR1^CreER/+^ mice via oral gavage, with a three-day interval between each dose. This dosage was repeated two additional times after seven days for the following two weeks, totaling 6 doses over three weeks. Eight days after the second dose, GL261 cells were implanted intracranially into one cerebral hemisphere of each mouse. After seventeen days, bioluminescent images were taken of the treated mice to observe GBM tumor formation.

### 2.5. Flow Cytometry

Perfusion with cold 1x PBS was performed, brains were isolated, and the area of the tumor or PBS injection site was removed. Single-cell suspensions were fixed using 10% formalin in PBS, cells were permeabilized using 2× saponin, and an Fc receptor blocker was added.

Antibodies for flow cytometry include: PerCP/Cy5.5 anti-mouse Siglec H (Biolegend; San Diego, CA, USA, 129614), APC anti-mouse CX3CR1 (Biolegend, San Diego, CA, USA; 149008), FITC F4/80 anti-mouse (Biolegend; 123108), PerCP/Cyanine5.5 anti-mouse/human CD11b (Biolegend, San Diego, CA, USA; 101228), APC anti-P2RY12 (Biolegend, San Diego, CA, USA; 848006), Alexa Fluor 488 TMEM119 monoclonal antibody (V3RT1GOsz) (ThermoFisher, Grand Island, NY, USA; 53-6119-82), and NF-κB p65 polyclonal antibody ALEXA FLUOR^®^ 555 Conjugated (Bioss Antibodies; Woburn, MA, USA; bs-0465R-A555). Flow cytometry was performed using a BD Biosciences Accuri C6 Flow Cytometer (San Jose, CA, USA). The data were analyzed using accompanying BD CSampler software, version 1.0.264.21.

### 2.6. Quantification of Tamoxifen Concentration in Mouse Brain Tissue via Liquid Chromatography with Tandem Mass Spectrometry (LC-MS/MS)

Cerebral brain tissue from tamoxifen-treated and corn oil-treated p65^fl/fl^ and p65^fl/fl^/CX_3_CR1^CreER/+^ mice was homogenized in Tris-HCl (pH 7.4; 1:2 *w/v*) using Potter-Elvehjem tissue grinders and a 60 Sonic Dismembrator sonicator (Fisher Scientific, Grand Island, NY, USA). Each homogenate was mixed with ten volumes of hexane:butanol (98:2 *v/v*), and the homogenates were resuspended. Three corn oil-treated brain homogenates and all the tamoxifen-treated brain homogenates were spiked with 1 mL of Tamoxifen-d_5_ internal standard (Cayman Chemicals; Ann Arbor, MI, USA, 18262) for quantification purposes. Each sample was vortexed and rotated for 1 h to extract the tamoxifen from the tissue. Samples were centrifuged at 13,200 rpm for 10 min, and the extraction was repeated on the pellets using 2 mL of hexane:butanol (98:2 *v/v*). The combined supernatants were dried using a vacuum centrifuge and reconstituted in 70% methanol. LC-MS/MS using a Triple Quad 6500+ system (SCIEX, Framingham, MA, USA) was performed on all samples and on a 1.35 nM tamoxifen standard dissolved in methanol to detect the presence of the quantifier ion (*m*/*z* = 72.08). Tamoxifen concentration was determined using a tamoxifen standard curve. GraphPad Prism 10 (Boston, MA, USA) was used for statistical analysis using a Student’s *t*-test.

### 2.7. Primary Bone Marrow-Derived Macrophages and Microglia Isolation

BMDMs were harvested and cultured using our previously published protocol [17]. BMDMs from p65^fl/fl^, p65^fl/fl^LysMCre, LysMCre, and CX_3_CR1^CreER^ mice served as controls for p65^fl/fl^CX_3_CR1^CreER/+^ microglia.

Primary microglia from tamoxifen-treated and corn oil-treated p65^fl/fl^LysMCre, CX_3_CR1^CreER^, p65^fl/fl^, and p65^fl/fl^CX_3_CR1^CreER/+^ mice were isolated according to our lab’s previously published protocol [19]. Genomic DNA was purified using the Monarch Genomic DNA Purification Kit (NEB, Ipswich, MA, USA; T3010S).

### 2.8. Optimization of PCR for Detecting Deletion of the p65 Gene

PCR primers were designed to bind upstream of *p65* exon 5 and downstream of exon 8 to determine tamoxifen-induced Cre deletion in microglia of the p65KO model [16]. Multiple PCR parameters were tested to optimize the protocol: 1× EmeraldAmp Max PCR Master Mix (TakaraBio; RR320A (Japan)), forward primer (IDT; 5’-GGACCAGGAACAGTTCGAATCTC-3’, 0.2 µM), reverse primer (IDT; 5’-CAATCCGGT GGCGATCATCTG-3′, 0.2 µM), sterile water, and genomic DNA template (150 ng).

The following PCR parameters were used for the LightCycler^®^ 96 (Roche, Basel, Switzerland): 1 denaturation cycle at 94 °C for 2 min, 35 cycles of denaturation at 94 °C for 20 s, annealing at 54.7 °C for 30 s, and elongation at 72 °C for 3 min. This was followed by 1 elongation cycle at 72 °C for 6 min. PCR products were examined on a 1% agarose gel to observe the intact *p65* gene band (about 6 kb) or the DNA band that indicates deletion of p65 exons 5–8 (about 4 kb). The LightCycler 96 SW 1.1 software was used to analyze the data (Roche, Basel, Switzerland).

### 2.9. Analysis of p65 in GBM Tumors or Microglia via Western Blot

Cerebral brain tissue from PBS-treated or GL261-Luc-implanted control, CX_3_CR1^CreER^, and p65^fl/fl^ mice were collected. In a separate experiment to determine p65 expression in microglia from treated p65KO and control mice, microglia from 4 tamoxifen-treated and 4 corn oil-treated p65KO mice and CX_3_CR1^CreER^ mice were harvested by following microglia harvest [19]. Microglia from four mice per group were pooled.

Cerebral brain tissue or microglia were processed for immunoblotting using our published procedures [17]. Proteins were separated on a 10% acrylamide resolving gel, and the nitrocellulose membranes were incubated overnight with primary antibodies for p65 (1:1000; CST; 8242) and β-actin (1:2000; CST; 4970) at 4 °C. Densitometry analysis was performed using ImageJ, version 1.53k (NIH, USA). Normalized p65/β-actin density ratios were calculated for each of the treated groups from the western blot using ImageJ and graphed with GraphPad Prism 10. p65 and loading control blots were exposed simultaneously under the same conditions.

### 2.10. Gene Expression Analysis of Microglia Co-Cultured with GL261 Cells

Mice were treated via oral gavage every other day for four days with 4mg/kg tamoxifen (*n* = 7), or corn oil as control (*n* = 7). Microglia were harvested five days after the last dose was administered using the Neural Tissue Dissociation Kit (P) (Miltenyi Biotec, Gaithersburg, MD, USA; 130-092-628) as previously described [19].

A total of 500,000 GBM GL261 cells in 1.5 mL of microglia media were plated on top of 0.4 µm polycarbonate transwell inserts (Corning, Corning, NY, USA, 07-200-165). Additionally, 966,000 primary microglia in 2.5 mL microglial media were plated in the bottom of the transwell dish. The plates were incubated at 37 °C/5% CO_2_ for 24 h, and microglia were then collected for RNA isolation using the RNeasy Micro Plus kit (Qiagen, Venlo, The Netherlands; 74034) and QIAshredder spin columns (Qiagen, Venlo, The Netherlands; 79654), according to the manufacturer’s directions. cDNA was produced using the SuperScript II kit (Thermo/Fisher, Grand Island, NY, USA; 11904018) according to manufacturer instructions.

Gene expression was analyzed in pooled microglia from p65KO mice. Groups were corn oil-treated mice (*n* = 3), tamoxifen-treated mice (*n* = 3), microglia from corn oil mice additionally co-cultured with GBM cells (*n* = 4), and microglia from tamoxifen-treated mice additionally co-cultured with GBM cells (*n* = 4).

Gene expression was analyzed via qPCR using TaqMan primer/probes (Thermo Fisher Scientific), 2× RT-PCR Mix (Roche 06402682001), and a Roche LightCycler 96 machine. Genes analyzed included the following: IL-1β (Thermo/Fisher, Grand Island, NY, USA; 4453320), Nos-2 (Thermo/Fisher, Grand Island, NY, USA; 4453320), Arg1 (Thermo/Fisher, Grand Island, NY, USA; 4453320), Mrc1 (Thermo/Fisher, Grand Island, NY, USA; 4448892), IL-10 (Thermo/Fisher, Grand Island, NY, USA; 4453320), and GusB (Thermo/Fisher, Grand Island, NY, USA; 4331182), which was used as an internal reference. Samples were run in triplicate. GraphPad Prism 10 software was used to generate graphs and analyze data via Student’s unpaired *t*-test (two-tailed); *p*-value of 0.05 or less. Exact *p*-values are indicated.

## 3. Results

### 3.1. Increased p65 Expression in Mice with GBM Tumors

**Figure 1:**
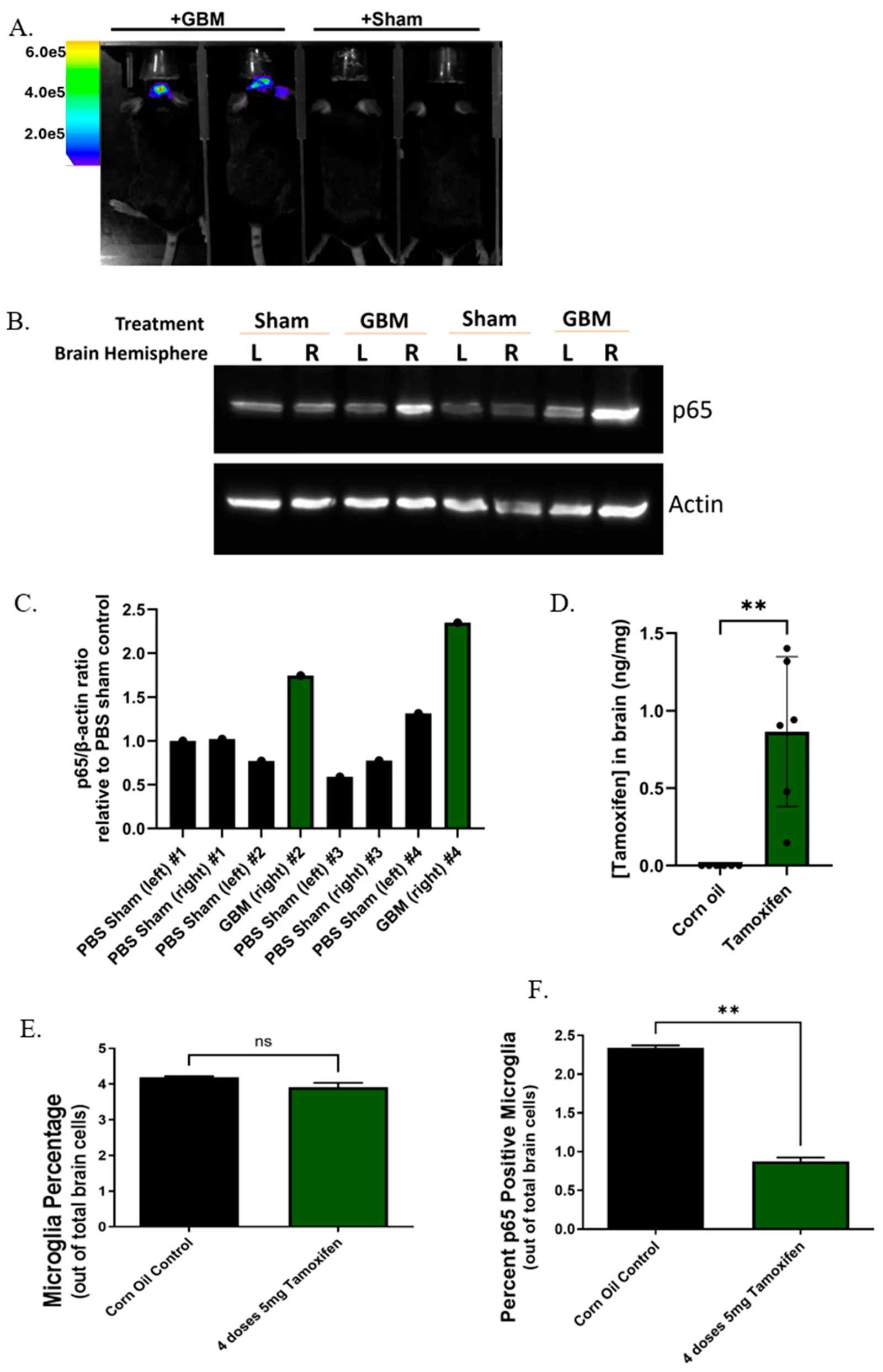
p65 expression in GBM tumors and microglial p65 expression in p65KO mice. (**A**) Representative bioluminescent images of control mice implanted with either GL261-Luc GBM cells or PBS sham into the right hemisphere. (**B**) Western blot and quantification analysis show increased p65 protein in GBM-implanted mice compared to sham mice (N = 2 per group). Sham mice received PBS injection into both the left and right hemispheres. GBM mice received GL261-Luc cells injected into the right brain hemisphere, and PBS injected into the left brain hemisphere. β-actin was used as a loading control. (**C**) Western blot quantification from (**B**). (**D**) Quantification of tamoxifen concentration in mouse brains via liquid chromatography with tandem mass spectrometry (LC-MS/MS). Tamoxifen concentration was calculated as the total amount of tamoxifen measured (in ng) per weight of the corresponding brain (in mg). A significantly higher tamoxifen concentration was measured in the brains of tamoxifen-treated mice compared to the brains of corn oil-treated mice (control group), which did not contain any detectable tamoxifen. Quantitative data shown are represented by mean ± SEM (*n* = 6 per group). A Student’s *t*-test was performed on the quantitative data (** *p* ≤ 0.01). (**E**) Flow cytometry on total brain cells to determine percent microglia in p65KO mice given corn oil or tamoxifen. Statistical analysis was performed in GraphPad Prism 10 using Welch’s *t*-test. (**F**) Flow cytometry on total brain cells to determine microglial p65 expression in p65KO mice given corn oil or tamoxifen. Statistical analysis performed in GraphPad Prism using Welch’s *t*-test. ** *p* = 0.0036. (**E**,**F**): corn oil-treated *n* = 3, tamoxifen-treated *n* = 2.

As high NF-κB signaling is associated with patient GBM tumor progression, we aimed to determine whether we would see this effect in our control mice that were implanted with GL261-Luc cells compared to PBS sham mice. Nineteen days post-implantation, GL261-Luc GBM tumors were visualized via bioluminescent imaging (Figure 1A). Western blotting indicated that p65 protein expression increased dramatically in mice that received GBM implantation compared to PBS sham controls (Figure 1B,C), indicating high canonical NF-κB signaling present in these tumors.

### 3.2. Generation of the p65^fl/fl^/CX_3_CR1^CreER/+^ Mouse Model

In our p65KO mouse model, tamoxifen must bind to the estrogen receptor found on the Cre recombinase within microglia for recombination activity to occur [15,20]. To confirm that the orally administered tamoxifen was able to penetrate the brains of the treated mice, tamoxifen was extracted from tamoxifen-treated or corn oil-treated p65^fl/fl^ and p65KO mice. Each mouse received a 4 mg/kg dose of either tamoxifen or corn oil via oral gavage for 3 consecutive days, and the brains were harvested the day following the last dose. LC-MS/MS was used to detect and quantify the concentration of tamoxifen in each brain. The quantifier ion used to detect tamoxifen in the extracted samples was the tamoxifen fragment ion with a mass/charge (*m*/*z*) ratio of 72.08. As expected, LC-MS/MS analysis showed no tamoxifen in the brains of the corn oil-treated mice (Figure 1D). In contrast, the mean tamoxifen concentration measured from the brains of the tamoxifen-treated mice was significantly higher (0.87 ng/mg of brain tissue) than in the corn oil-treated mice, confirming that the tamoxifen administered to mice via oral gavage can be extracted from brain tissue and quantified.

### 3.3. Reduced p65 Expression in Microglia Observed in Tamoxifen-Treated p65KO Mice

Flow cytometry was used to determine whether tamoxifen treatment would affect the number of microglia in the brain and to gauge Cre recombinase efficiency in microglia from tamoxifen-treated versus corn oil-treated 5-week-old p65KO mice. The percentages of microglia from corn oil-treated and tamoxifen-treated p65KO mice were similar, suggesting that these treatments do not notably change cell numbers (Figure 1E). However, p65 expression in microglia was significantly reduced in tamoxifen-treated p65KO mice compared to corn oil-treated mice (Figure 1F and Supplemental Table S1). This result demonstrates that *p65* gene recombination occurred in microglia from tamoxifen-treated p65KO mice; however, it suggests that not every microglia experienced recombination. Microglia were distinguished from other cell types using specific F4/80, Siglec-H, and CX3CR1 markers [19].

### 3.4. p65KO Microglia Exhibit Partial Deletion of the p65 Gene Following Tamoxifen Administration

**Figure 2:**
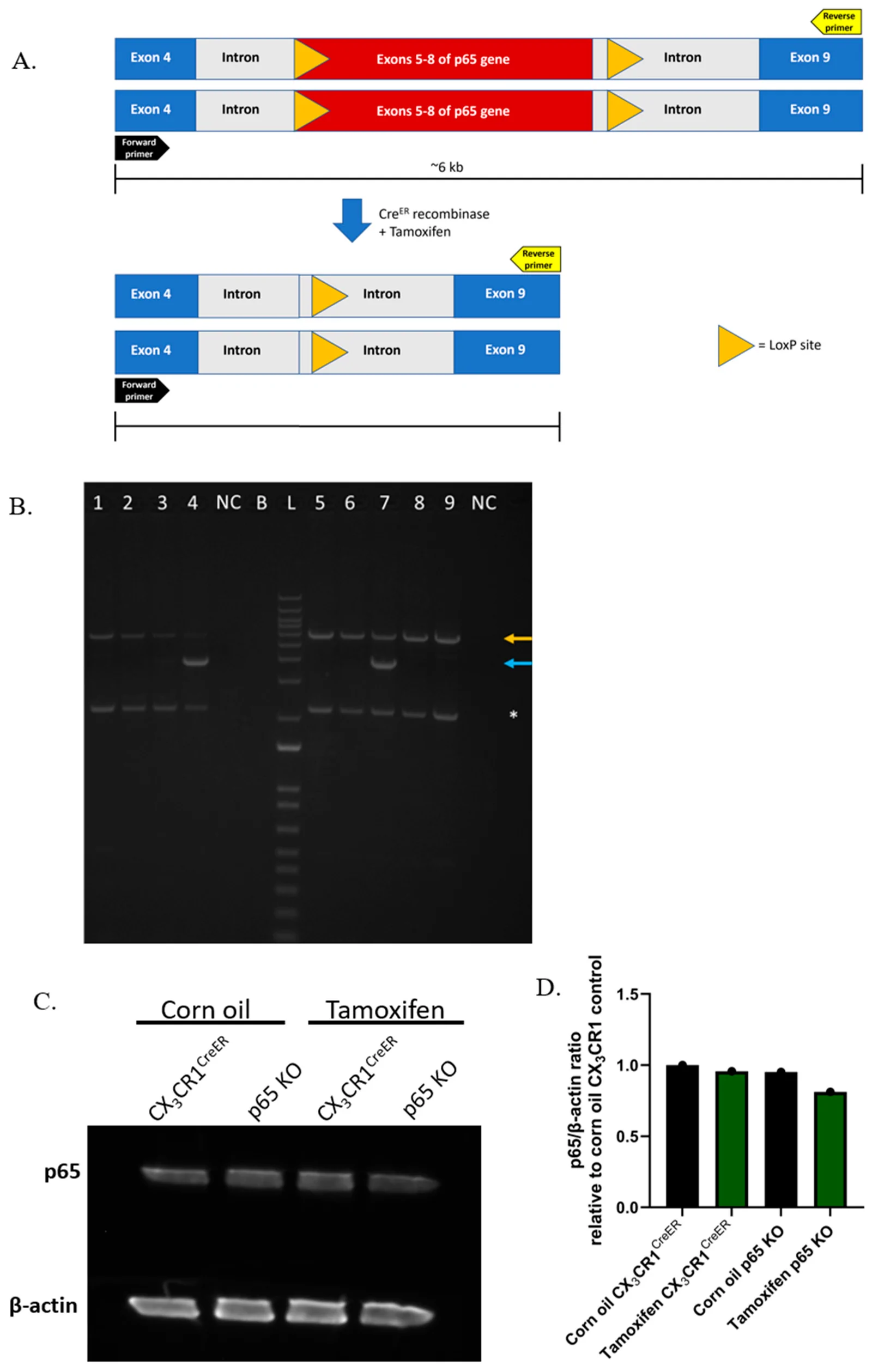
Partial *p65* gene deletion and decreased p65 protein expression in microglia from p65KO mice. (**A**) Deletion of exons 5–8 of the *p65* gene. This diagram depicts how the *p65* gene in microglia and bone marrow-derived macrophage (BMDM) genomic DNA should be altered following Cre recombination in mice that have a floxed *p65* gene [16]. (**B**) Partial deletion of the *p65* gene in microglia and BMDMs from mice. Microglia were isolated from corn oil- or tamoxifen-treated mice, whereas BMDMs were isolated from untreated mice. The top orange arrow indicates the intact *p65* gene (~6 kb), whereas the bottom blue arrow indicates the deletion of p65 exons 5–8 (~4 kb). A smaller PCR product about 2.2 kb in size (*) may be a nonspecific PCR product that was amplified. Legend: (1) corn oil p65^fl/fl^ microglia, (2) tamoxifen p65^fl/fl^ microglia, (3) corn oil p65KO microglia, (4) tamoxifen p65KO microglia, (5) p65^fl/fl^ BMDM, (6) LysMCre BMDM, (7) p65^fl/fl^LysMCre BMDM, (8) CX_3_CR1^CreER^ BMDM, (9) p65KO BMDM. “NC” signifies the negative control (no DNA), and “B” signifies that this well was skipped. A 1 kb+ DNA ladder was used (L). (**C**) Western blot of p65 protein levels in microglia isolated from treated control and p65KO mice. Microglia from tamoxifen-treated p65KO mice exhibited slightly lower p65 protein expression compared to controls. β-actin was used as a loading control. *n* = 4 mice per group (pooled microglia). (**D**) Image J quantification of p65 protein levels in microglia from the western blot (**C**).

To further determine the efficiency of *p65* gene deletion in microglial DNA from the tamoxifen-treated p65KO mice, PCR primers were designed to flank exons 5–8 of the *p65* gene, which is within the floxed region of this gene [16] (Figure 2A). Following treatment with tamoxifen, exons 5 through 8 should be deleted in p65KO mice, resulting in a shorter DNA band that indicates *p65* gene deletion; the deletion product should be ~4 kb, whereas the expected DNA band representing the intact *p65* gene is approximately 6 kb.

As anticipated, microglia isolated from corn oil- and tamoxifen-treated p65^fl/fl^ control mice (lanes 1 and 2) displayed an intact *p65* gene since they do not express Cre recombinase (Figure 2B). However, microglia isolated from the tamoxifen-treated p65KO mouse (lane 4) exhibited deletion of the *p65* exons compared to the corn oil-treated p65KO mouse (lane 3) and treated p65^fl/fl^ mice. Bone marrow-derived macrophages (BMDMs) served as a control group for the microglia, and the BMDMs from untreated LysMCre, p65^fl/fl^, CX_3_CR1^CreER^, and p65KO mice (lanes 5, 6, 8, and 9, respectively) all exhibited an intact *p65* gene (Figure 2B). This PCR was also able to detect p65 exon excision in BMDMs from the p65^fl/fl^LysMCre mouse model, which was expected because active Cre recombinase is expressed under the LysM promoter to induce *p65* gene deletion even without tamoxifen treatment. In the microglia (and BMDMs) that exhibited p65 exon excision, the DNA band representing the intact *p65* gene was still observed, but at a lower concentration. This indicated that there was a partial deletion of the *p65* gene rather than complete deletion in those samples.

### 3.5. Slightly Decreased p65 Protein in Microglia from Tamoxifen-Treated p65KO Mice Corresponds to Partial Deletion of the p65 Gene

Since partial deletion of the *p65* gene was observed in microglia from tamoxifen-treated p65KO mice, it was next determined whether there was a corresponding decrease in p65 protein levels in these microglia. A western blot was performed to compare the relative microglial p65 protein in each of the following groups: corn oil-treated CX_3_CR1^CreER^, corn oil-treated p65KO, tamoxifen-treated CX_3_CR1^CreER^, and tamoxifen-treated p65KO mice (Figure 2C,D). The corn oil-treated CX_3_CR1^CreER^ mice served as a base control group to compare with the other three groups to determine the percent change in p65 protein expression. The amount of p65 protein in microglia from the corn oil-treated p65KO and tamoxifen-treated CX_3_CR1^CreER^ control mice decreased by 4.77% and 4.29%, respectively, compared to the amount of p65 protein in microglia from the corn oil-treated CX_3_CR1^CreER^. In contrast, the amount of p65 protein from the tamoxifen-treated p65KO mouse microglia decreased by 18.79% compared to that of the corn oil-treated CX_3_CR1^CreER^ mice, indicating that there was slightly less p65 protein in the microglia from the tamoxifen-treated p65KO mice, which was consistent with our earlier partial *p65* gene deletion results.

### 3.6. Determining Microglia p65 Expression in Treated p65KO Mice Implanted with GBM Cells

**Figure 3:**
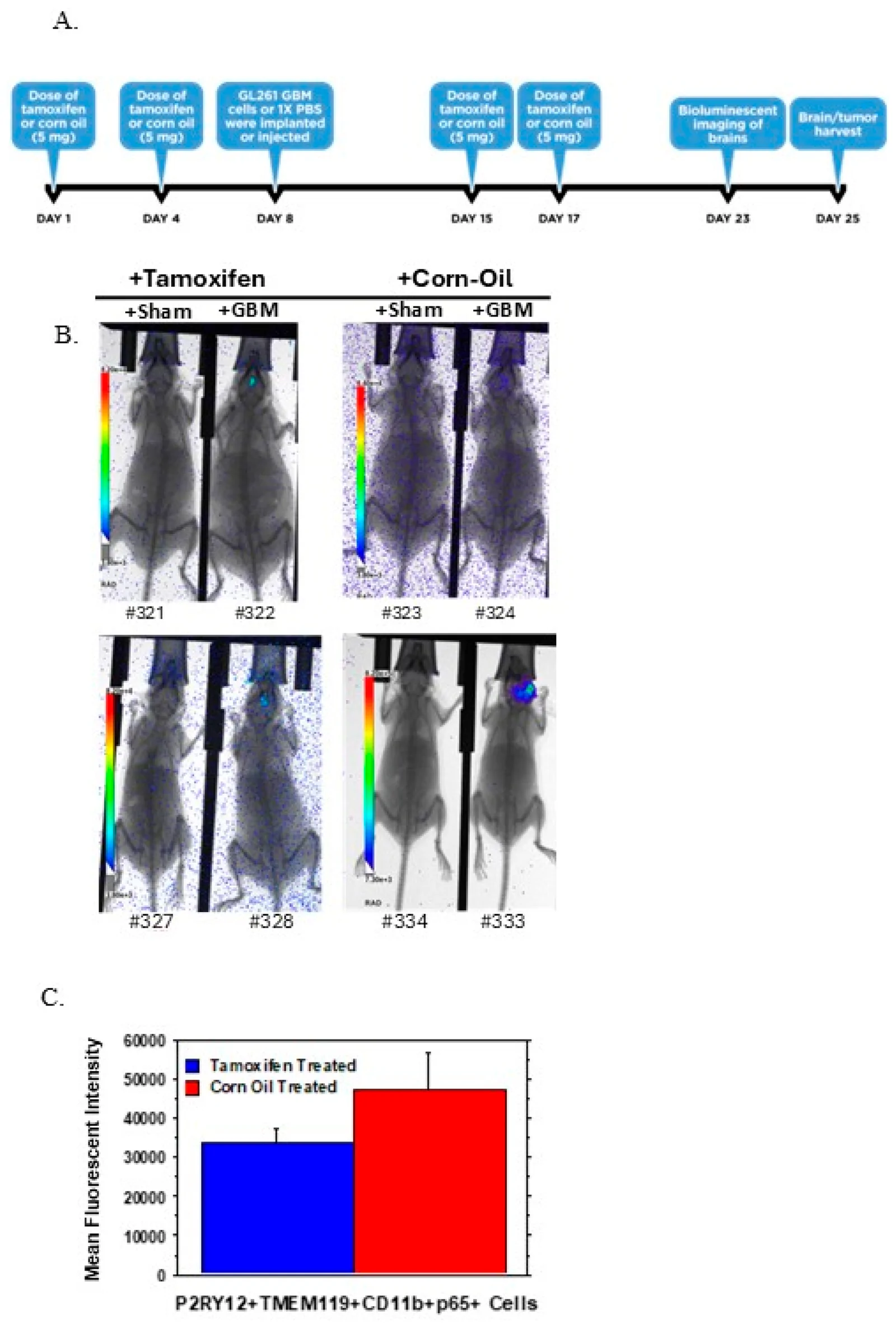
p65 deletion in microglia of corn oil- or tamoxifen-treated mice implanted with GBM tumors. (**A**) Timeline for the treatment schedule, orthotopic GL261 implantation, and harvest for flow cytometry. Two doses of treatment (corn oil or tamoxifen) were administered orally before and after GL261-Luc implantation or PBS sham injection. (**B**) Bioluminescent images of GBM tumors in mice prior to flow cytometry. Four of the eight mice (322,324,328,333) received GL261-Luc implantation. The four remaining mice (321,323,327,334) received PBS sham injections into the brain. (**C**) p65 fluorescence intensity in microglia of treated or control p65KO mice implanted with GBM tumors (322,324,328,333). Compared to the corn oil-treated mice, the tamoxifen-treated mice exhibited lower p65 fluorescence intensity, which corresponds to lower p65 expression within the identified microglia, *p* = 0.3069 (*n* = 2 per group).

We also aimed to evaluate how tamoxifen administration would affect microglial p65 in the context of implanted GBM tumors. p65KO mice were given two tamoxifen or corn oil doses before GL261-Luc GBM implantation or PBS injections and two doses following injection (Figure 3A). The mice were imaged using bioluminescence to verify the presence or absence of GBM tumors (Figure 3B). Tumors in the corn oil-treated GBM mice looked visually larger than the tumors of the tamoxifen-treated GBM mice via bioluminescent imaging. The GBM tumor region or brain tissue where the PBS was injected was harvested, and flow cytometry was used to determine if there was a lower expression of p65 in microglia in the tamoxifen-treated p65KO mice compared to corn oil controls. The specific markers used to identify microglia were CD11b, P2RY12, and TMEM119 [7]. This set of markers will identify both resting and activated microglia, as P2RY12 and TMEM119 are often downregulated in microglia, but not absent. The microglial subpopulation was identified from the entire population of brain cells and debris from each brain sample by first gating for P2RY12+ cells. Of those P2RY12+ cells, only those positive for both CD11b and TMEM119 were analyzed because these triple-positive cells represent the microglial population. p65 expression was then analyzed in this microglia subpopulation (Figure 3C). Microglial p65 expression in the tamoxifen/GBM mice was lower than in the corn oil/GBM mice (Figure 3C). Overall, these results were consistent with our previous results showing partial microglial *p65* gene deletion in tamoxifen-treated p65KO mice. A longer treatment period of six doses of tamoxifen was also performed, but the data did not show additional p65 knockdown compared to controls (Supplemental Figure S1 and Figure S1 Text file).

### 3.7. Partially Deleted NF-κB Increases M2 Gene Expression in Microglia Co-Cultured with GL261 GBM Cells

**Figure 4:**
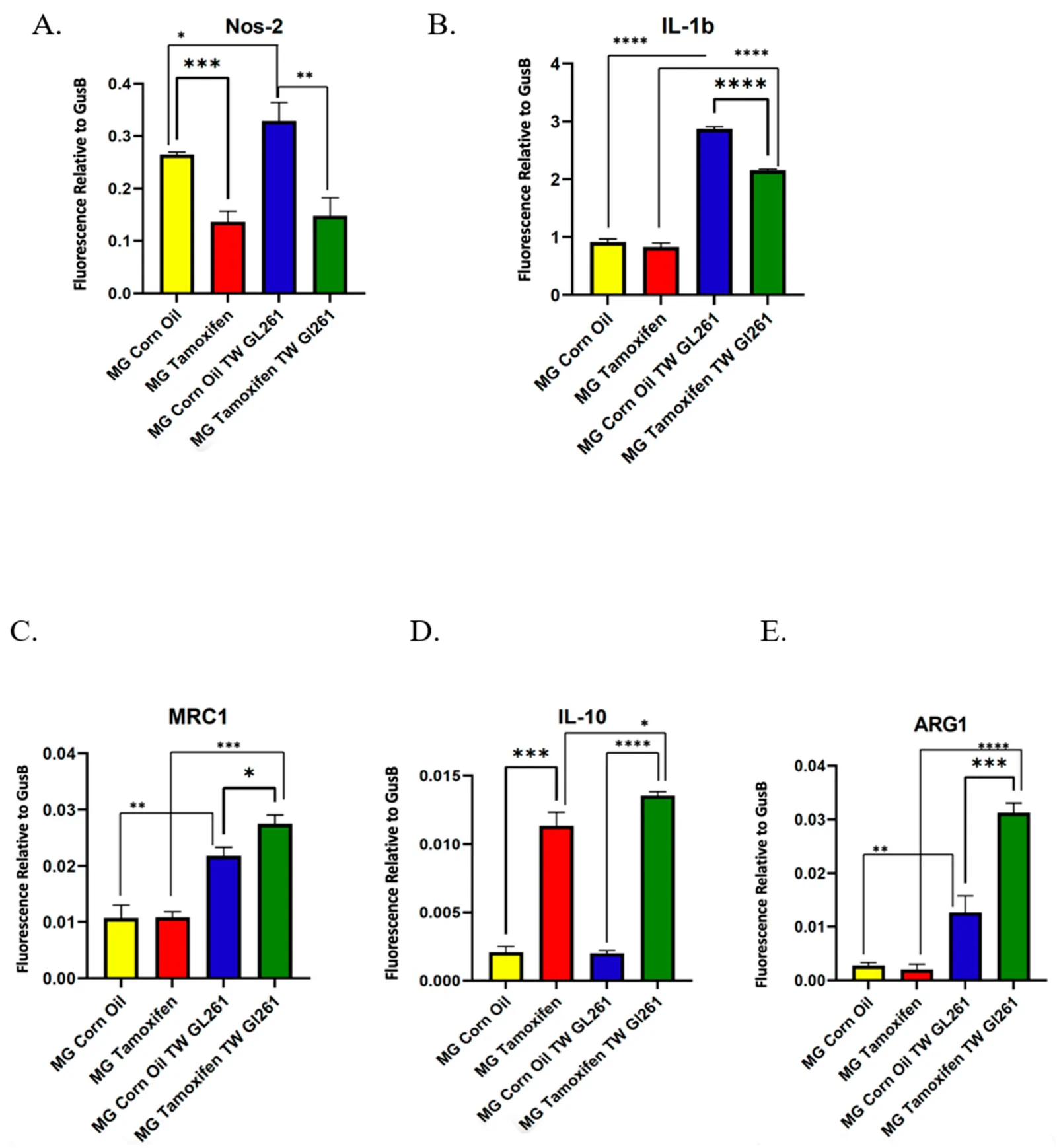
Partially deleted NF-κB increases M2 gene expression in treated primary microglia co-cultured with GL261 GBM cells. Gene expression was analyzed in pooled microglia from p65KO mice. Groups were corn oil-treated mice (*n* = 3), tamoxifen-treated mice (*n* = 3), microglia from corn oil-treated mice additionally co-cultured with GBM cells (*n*=4), and microglia from tamoxifen-treated mice additionally co-cultured with GBM cells (*n* = 4). M1 primers included Nos-2 (**A**) and IL-1β (**B**). M2 primers included Mrc1 (**C**), IL-10 (**D**), and Arg1 (**E**). GusB was used as the reference gene. A Student’s unpaired *t*-test (two-tailed) was used to determine the significance of fluorescence differences between the samples. *p* values are IL-1β **** < 0.0001; Nos-2 *** = 0.0005, ** = 0.0031, * = 0.0338; Mrc1 *** = 0.0001, ** = 0.0023, * = 0.0113; IL-10 **** < 0.0001, *** = 0.0001, * = 0.0206; Arg1 **** < 0.0001, *** = 0.0009, ** = 0.0055. Samples were run in triplicate.

Although we observed only modest decreases in p65 deletion in our p65KO microglia using PCR and immunoblotting of p65 in isolated primary microglia, we sought to determine if even small alterations in NF-κB signaling could affect microglial phenotype polarization, and whether in vitro culture with GBM cells exacerbated these changes. In response to certain environmental conditions or factors, microglia can polarize to different phenotypes, which include anti-tumor/pro-inflammatory (M1) or tumor-promoting/anti-inflammatory (M2) states [4]. To observe how the inhibition of NF-κB signaling affects the polarization state of microglia in the presence of GBM cells, quantitative PCR was performed to evaluate expression levels of various M1 and M2 phenotype markers from microglia that were cultured in the presence or absence of GL261 syngeneic GBM cells. Four different groups of microglia were evaluated for M1 and M2 marker expression levels: microglia from (1) corn oil- and (2) tamoxifen-treated p65KO mice, and microglia from (3) corn oil and (4) tamoxifen-treated p65KO mice that were co-cultured with GL261 GBM cells. Nos-2 and IL-1ꞵ are typical M1 markers, whereas Mrc1, IL-10, and Arg1 are M2 markers. The expression levels of Nos-2 and IL-1ꞵ were significantly lower in microglia from tamoxifen-treated p65KO mice that were co-cultured with GL261 cells compared to the co-cultured microglia from vehicle-treated p65KO mice (Figure 4A,B). Nos-2 was also significantly decreased in microglia with p65 knocked down (Figure 4A). In contrast, the expression levels of Mrc1, IL-10, and Arg1 were significantly higher in microglia from tamoxifen-treated p65KO mice that were co-cultured with GL261 cells compared to the co-cultured microglia from vehicle-treated p65KO mice (Figure 4C–E). Interestingly, IL-10 expression significantly increased in microglia from p65KO mice. These results suggest that NF-κB may be needed to promote an M1 phenotype in microglia grown in the presence of GL261 cells. Even with slightly decreased NF-κB signaling, microglia co-cultured with GL261 GBM cells were pushed towards a more M2 phenotype. Additional work with complete NF-κB deletion is needed to confirm these results.

## 4. Discussion

GBM remains one of the most lethal cancers as it still lacks an effective treatment regimen to reduce tumor growth and overcome drug resistance [1,3]. Targeting both tumor cells and components of the GBM TME that exert pro-tumor effects is most likely necessary to inhibit tumor development [21]. Hyperactive canonical NF-κB signaling has been implicated in promoting GBM tumor growth, drug resistance, and microglia/macrophage polarization. Thus, studying how inhibition of NF-κB in the GBM TME impacts GBM tumors can offer practical knowledge for the development or repurposing of current GBM drugs [4,10,11,22,23]. Currently, there is inadequate information about how canonical NF-κB signaling in TME immune cells impacts tumors [21], so knowledge obtained from studying this pathway in mouse models would be beneficial for improving patient outcomes.

Multiple NF-κB mouse models have been generated and reported for their use in studying how NF-κB signaling in the tumor microenvironment plays a role in specific diseases and conditions [16,24,25]. These models involve overexpression or knockout of an NF-κB pathway member, which includes the receptors involved in triggering the NF-κB pathway, IKK complex members, and NF-κB transcription factors like p50 [24,25]. These NF-κB knockout mouse models often apply the Cre-loxP system to induce cell type-specific deletion of the desired gene [26,27,28]. In particular, the CreERT-loxP system is suitable for creating conditional and inducible mouse models and is particularly useful for knocking out genes that are required for proper mouse development, such as *p65*, which is the gene that encodes the NF-κB p65 transcription factor [15,16,20]. Mice that exhibit systemic p65 deletion succumb to embryonic lethality due to liver degeneration from hepatocyte apoptosis [16]. Therefore, having the ability to induce deletion of the *p65* gene in a particular cell type at any time point following development is key to generating viable p65 knockout mice that can be used in various studies.

This study aimed to characterize a tamoxifen-inducible p65^fl/fl^/CX_3_CR1^CreER/+^ transgenic mouse model to determine if the *p65* gene was efficiently deleted in microglia. In this model, canonical NF-κB signaling should be inhibited due to the deletion of *p65* exons 5 through 8, which encode most of the Rel homology domain [16]. Without this domain, the truncated p65 protein cannot form proper homo/heterodimers or bind to the DNA, thus preventing downstream NF-κB signaling [16]. Based on our results, we demonstrate that elevated levels of p65 are found in GBM tumors in mice (Figure 1B), which recapitulates what is seen in human patients. Orally administered tamoxifen can penetrate the mouse brain, which was confirmed by LC-MS/MS analysis on the brains of tamoxifen-treated mice compared to vehicle-treated mice (Figure 1D). After confirming that tamoxifen does reach the brain (Figure 1D), we assessed the deletion of exons 5 through 8 in microglia from tamoxifen-treated p65KO and control mice. Both the partial deletion of the floxed *p65* gene and decreased microglial p65 protein from tamoxifen-treated p65-deficient mice suggest that the excision of the floxed *p65* gene in microglia was incomplete but not entirely absent (Figure 2B,D). These results cannot distinguish whether only a subset of the microglial population exhibited *p65* gene deletion, or whether there was incomplete deletion across the microglial population, or whether both scenarios occurred. The results also cannot rule out whether other cells of similar size to microglia were accidentally isolated with the microglia, which would harbor a normal *p65* gene. There was a nonspecific band of ~2.2 kb that appeared in every cellular sample despite multiple experimental adjustments. A Primer-BLAST search against the *Mus Musculus* genome did not indicate the primers would bind to a homologous region outside the target. Additional analysis, such as sequencing the band, would provide insight into why it was consistently amplified.

The variability in the amount of decreased p65 expression seen in the GBM flow cytometry experiments also supports the idea of partial deletion of the *p65* gene (Figure 3 and Supplemental Figure S1). Further flow cytometry experiments using more mice would be needed to account for variability in the p65 fluorescence intensity values. Further studies could use different microglial markers such as Siglec-H, F4/80, and CX3CR1, which are also highly specific for microglia. Potential sex differences in gene deletion efficiency could contribute to the variation in p65 expression and could correspond to differences in tumor sizes between treated male and female mice (Figure 3). Mouse variation and tamoxifen or corn oil administration could also be potential reasons for the variation seen in p65 fluorescence intensity values among these treated mice.

Our gene expression analysis was interesting considering many studies have found that hyperactive canonical NF-κB signaling promotes M1 to M2 macrophage polarization [4,10,11,22,23]. Microglia with partially deleted p65 that were co-cultured with syngeneic GL261 GBM cells were found to have elevated levels of M2 markers Mrc1, IL-10, and Arg1, and decreased M1 markers Nos-2 and IL-1β as compared to control cells cultured with GBM cells. (Figure 4). In this cell culture context, NF-κB may be needed to prevent or slow polarization to an M2 phenotype in GBM TME microglia. Further studies are needed to build on these preliminary findings, such as microglial functional assays using fully p65-deficient cells. This would be important in confirming whether the microglial cells with deficient NF-κB signaling exhibit M2 phenotype behavior in the context of GBM cells. This is necessary as our data with partial p65 deletion is counter to other p65KO models targeting macrophages. This information could be important in developing future GBM treatment options.

Multiple NF-κB mouse models have been generated to study NF-κB signaling in various diseases and conditions [16,24,25], but as other studies and the current model demonstrate, deletion of NF-κB in microglia seems to be especially challenging. One study employed a LysM-Cre/Ikkβ^F/F^ mouse model in which microglia and other myeloid cells are IKKβ-deficient due to Cre-mediated recombination of the floxed *Ikkβ* gene [26]. Their study noted that cultured primary microglia from neonatal LysM-Cre/Ikkβ^F/F^ mice exhibited partial deletion (36%) of the *Ikkβ* gene, as determined by RT-PCR analysis [26]. After observing low *Ikkβ* deletion (4%) in microglia from adult transgenic mice, they found that LPS stimulation in adult mice increased microglial *Ikkβ* deletion to 31% [26]. This suggests that we may need to stimulate canonical NF-κB signaling in microglia from our tamoxifen-treated, adult p65KO mice to observe greater deletion of the gene and ablation of the p65 protein. In another study, Steinbrecher et al. generated intestinal epithelial cell-p65KO mice using the Cre-loxP system and demonstrated loss of p65 protein in isolated primary colonocytes from their KO mice [16]. In another study, Zhang et al. also used the p65^fl/fl^ mice from the A.S. Baldwin lab [16], but crossed them with mice that express Cre recombinase under the α-myosin heavy chain promoter to generate cardiomyocyte-specific p65KO mice [27]. Unlike our results, they noted an almost complete absence of p65 protein in the isolated cardiomyocytes from their p65KO model [27]. Lastly, Ijaz et al. generated a fibroblast-specific p65-deficient mouse model using their own p65^fl/fl^ mouse, in which exons 5 through 8 of the *p65* gene were deleted following intraperitoneal tamoxifen administration [28]. Both p65 mRNA and protein expression levels in isolated fibroblasts from these mice decreased by about 50% compared to control mice that lacked Cre recombinase expression [28]. Like our model, two of these NF-κB knockout mouse models exhibited only partial ablation of either IKKβ or p65 protein [26,28]. This all raises the question of why targeting the NF-κB pathway in certain cell types, like microglia, is more difficult than in other cell types.

There are several important limitations of this study. Initially, 50 µL of 1 mg/mL^−1^ tamoxifen was administered by injection into the stomachs of pups as young as postnatal day 1, which had previously been shown [29]. This method was quickly discontinued, however, due to 100% lethality, which is why older mice were then treated with tamoxifen via oral gavage [30]. While multiple tamoxifen treatment regimens were conducted, including treating neonates and adults with various tamoxifen concentrations for varying lengths of time and by two different treatment routes, we recognize that not every tamoxifen treatment avenue was exhausted, such as feeding pregnant females tamoxifen chow. Measuring the expression of Cre recombinase in microglia from both vehicle- and tamoxifen-treated CX_3_CR1^CreER^ control and p65KO mice might be necessary to determine if unequal Cre recombinase expression among the different mouse genotypes contributed to the partial gene deletion observed in the tamoxifen-treated p65KO mice [31].

Similar to our p65KO mouse model, one study generated an adipocyte-specific p65 knockout mouse model in which exons 2 through 4 were excised, and both mRNA and protein expression of p65 were reduced, as well as the expression of downstream target genes [32]. Thus, testing the deletion of various groups of exons within the *p65* gene may be necessary to determine which exons are essential to delete for complete p65 ablation in microglia, as this requirement may differ between different cell types.

Due to the trial nature of the study, it is also important to note the small numbers of animals used in each condition. The p65KO model were poor breeders, which led to challenges in obtaining large numbers of experimental animals. While we did see significant differences in microglial gene expression in treated primary microglia co-cultured with GL261 GBM cells (Figure 4), it is important to note the preliminary nature of these findings. Further work is needed to fully connect the role of p65 deletion in GBM tumor-associated microglia polarization.

## 5. Conclusions

In conclusion, this study’s aim was to develop a microglia-specific p65KO mouse model, but after characterization, it was determined that only minimal deletion of the floxed *p65* gene in microglia was achieved. Minimal p65 deletion in microglia led to an increase in M2 gene expression in microglia co-cultured with GL261 GBM cells. This preliminary result indicates the importance of future investigation into canonical NF-κB signaling in GBM-associated microglia, as these native CNS cells might be a viable GBM therapy target.

## Data Availability

The raw data supporting the conclusions of this article will be made available by the authors upon request.

## Supplementary Materials

The following supporting information can be downloaded at: https://www.mdpi.com/article/10.3390/cells15181668/s1, Figure S1: Second flow cytometry to test a longer treatment schedule. (A) Timeline for the treatment schedule, orthotopic GL261-Luc implantation, and harvest for the second flow cytometry trial. For a longer tamoxifen treatment administration, three rounds (six doses total) of tamoxifen or corn oil were given via oral gavage to the mouse before GL261-Luc implantation. (B) Histograms of p65 expression in microglia for the second flow cytometry experiment. p65 expression was measured by p65 fluorescence intensity. (C) p65 fluorescence intensity of treated p65 KO mice from the second flow cytometry experiment. Three male p65 KO mice were given GBM tumors and either received tamoxifen or corn oil before implantation. One tamoxifen-treated mouse had a higher p65 expression compared to the corn oil-treated mouse; Table S1: Percentage of Microglia and p65+ Microglia from p65 KO Mice. Figure S1 Text file: Supplemental Figure S1 Information.
