# Supplementary material for: Challenges in Developing a Microglial NF-Kappa B Conditional Mouse Model for Glioblastoma Studies
Source: Cells. 2026 Sep 16;15(18):1668. doi: 10.3390/cells15181668 (PMC13605159; doi:10.3390/cells15181668)
Supplement: Supplementary file 1 [file cells-15-01668-s001.zip › Figure S1 Text file.pdf]

Supplemental Figure S1 Information

Methods

The second treatment schedule involved longer tamoxifen or corn oil administration to p65 KO, $p65^{fl/fl}$ control, and CX_3CR1^{CreER} control mice prior to GBM implantation. Two doses of 5 mg tamoxifen or corn oil control were administered to three $p65^{fl/fl}CX_3CR1^{CreER/+}$ mice via oral gavage, with a three-day interval between each dose. This dosage was repeated two additional times after seven days for the following two weeks, totaling 6 dosages in a span of three weeks. Eight days after the second dose, GL261 cells or 1X PBS were implanted intracranially or injected into one cerebral hemisphere of each mouse. After seventeen days, bioluminescent images were taken of the treated mice to observe for GBM tumor formation using the Ami X imaging system.

Results

To test if a longer treatment period would affect p65 expression in microglia, six doses of treatment (tamoxifen or corn oil) were given to male p65 KO mice before GBM implantation (**Supplemental Fig. S1**). The microglia were identified, and p65 expression was analyzed as explained in the main text. The p65 fluorescence intensity values were inconsistent with the expected result as there was a tamoxifen-treated GBM mouse that showed higher p65 expression compared to the corn oil-treated GBM mouse. Based on both flow cytometry experiments, it appeared that the tamoxifen treatment was inconsistent in inducing deletion of the *p65* gene in microglia associated with GBM tumors. There was lower p65 expression in some tamoxifen/GBM mice compared to the corn oil/GBM mice, but this was not observed in every tamoxifen/GBM p65 KO mouse.

Supplemental 1 Figure Legend. Second flow cytometry to test a longer treatment schedule.

(A) Timeline for the treatment schedule, orthotopic GL261-Luc implantation, and harvest for the second flow cytometry trial. For a longer tamoxifen treatment administration, three rounds (six doses total) of tamoxifen or corn oil were given via oral gavage to the mouse before GL261-Luc implantation. **(B)** Histograms of p65 expression in microglia for the second flow cytometry experiment. p65 expression was measured by p65 fluorescence intensity. **(C)** p65 fluorescence intensity of treated p65 KO mice from the second flow cytometry experiment. Three male p65 KO mice were given GBM tumors and either received tamoxifen or corn oil before implantation. One tamoxifen-treated mouse had a higher p65 expression compared to the corn oil-treated mouse.
