# Supplementary material for: Challenges in Developing a Microglial NF-Kappa B Conditional Mouse Model for Glioblastoma Studies
Source: Cells. 2026 Sep 16;15(18):1668. doi: 10.3390/cells15181668 (PMC13605159; doi:10.3390/cells15181668)
Supplement: Supplementary file 1 [file cells-15-01668-s001.zip › Table S1 final.pdf]

Supplemental Table S1. Percentage of Microglia and p65+ Microglia from p65 KO Mice.

	% of total brain population				
	Given corn oil			Given 4 doses of 5 mg tamoxifen	
	Male	Female	Female	Male	Female
F4/80, CXCR1, SiglecH	4.16	4.21	4.21	3.82	4
F4/80, CXCR1, SiglecH, p65	2.31	2.36	2.36	0.84	0.91
